# Pediatic code blue event anaylsis: Performance of non-acute health-care providers

**DOI:** 10.1080/10872981.2022.2106811

**Published:** 2022-08-01

**Authors:** Graham Chamberlain, Ronish Gupta, Anna-Theresa Lobos

**Affiliations:** a Department of Pediatrics CHEO, Ottawa, Ontario, Canada; b Department of Pediatrics, McMaster University, Hamilton, Ontario, Canada; c School of Education, Johns Hopkins University, Baltimore, MD, USA; d Division of Critical Care, Department of Pediatrics, CHEO, Ottawa, Ontario, Canada

**Keywords:** Medical education, resuscitation, cardiopulmonary arrest, pediatrics, In-Hospital arrest, basic life support, hospital medicine, critical care

## Abstract

In-hospital pediatric cardiopulmonary arrest is rare. With more than 50% of patients not surviving to discharge following cardiopulmonary arrest, it is important that health-care providers (HCPs) respond appropriately to deteriorating patients. Our study evaluated the performance of basic life support skills using non-acute HCPs during pediatric inpatient resuscitation events. We conducted a retrospective chart review of all code blue team (CBT) activations in non-acute care areas of a tertiary care children’s hospital from 2008 to 2017. The main outcomes were frequency of life support algorithmic assessments and interventions (critical actions) performed by non-acute HCPs prior to the arrival of CBT. CBT activation and outcome data were summarized descriptively. Logistic regression was used to assess for an association of outcomes with the presence of established leadership. A total of 60 CBT activations were retrieved, 48 of which had data available on isolated non-acute HCP performance. Most children (93%) survived to discharge. Critical action performance review revealed that an airway, breathing and pulse assessment was documented to have occurred in 33%, 69% and 29% of cases, respectively. A full primary assessment was documented in 6% of cases. The presence of established leadership was associated with the performance of a partial ABC assessment. Our results suggest that resuscitation performance of pediatric inpatient non-acute HCPs often does not adhere to standard life support guidelines. These results highlight the need to reconsider the current approaches used for non-acute HCP resuscitation training.

## Introduction

In-hospital pediatric cardiopulmonary arrest (IHCA) occurring outside of acute care settings is an uncommon but potentially devastating event [1–4]. While survival following the IHCA has improved, more than 50% of children do not survive to discharge[2]. In children, cardiopulmonary arrest rarely occurs without signs of preceding decompensation[5]. Where possible, early recognition of deteriorating patients and intervention by non-acute health-care providers (HCPs) is critical in improving outcomes [6,7]. When faced with an acutely unstable situation; however, a robust knowledge of resuscitation guidelines is associated with improved resuscitation skills[8].

Prior to the arrival of critical care support, non-acute care providers on in-patient pediatric wards must provide basic life supports, such as chest compressions and pulse checks, when patients deteriorate. Available data from simulation studies have shown that non-acute pediatric HCPs, including both nurses and physicians, frequently deviate from these and other actions outlined in standard life support algorithms [9,10]. Resuscitation errors observed in simulation include both advanced life support skills and basic skills, such as pulse checks and simple airway maneuvers [9]. Moreover, these errors occurred despite health-care providers receiving standard training in both basic and advanced life support[10].

While this data comes from the simulation literature, it has been suggested in some studies that there is a correlation between resuscitation performance in simulation and in real-life settings[11]. To our knowledge, there are no studies examining the basic and advanced life support actions performed by non-acute HCPs in real-life pediatric resuscitations prior to the arrival of the code blue team (critical care-trained HCPs). This retrospective chart review was designed to explore what critical actions are performed by non-acute HCPs in real-life pediatric resuscitations before the code blue team arrives. Specifically, we were interested in the adherence to basic and advanced life-support guidelines. Based on the literature from simulation studies, we hypothesized that adherence to life support algorithms is not commonly achieved by non-acute care HCPs.

## Methods

### Study design and setting

This was a retrospective observational study at an academic tertiary care pediatric hospital in Canada. The project was deemed not to require review by the hospital research ethics board. Code blue activations between 1 January 2008 and 30 June 2017 were identified and reviewed for the current study. Code blue activations are expected for cardiac or respiratory arrest, life-threatening emergencies (e.g., severe sepsis, anaphylaxis, raised intracranial pressure), and intubations required outside a critical care area. Non-acute care providers (such as ward nurses) are required to maintain certification in American Heart Association (AHA) basic pediatric life support training and receive other acute-care education taught by educators (nurse or respiratory therapist) specific to their area of work. Pediatric resident trainees are required to be certified in both AHA Basic Life Support (BLS) and AHA Pediatric Advanced Life Support (PALS) and are provided certification and re-certification by the training program as needed. The hospital supports a simulation program, which provides regular mock code blue experiences in addition to the didactic small-group sessions provided by nursing educators on a variety of topics throughout the year. Members of the institutional code blue team include senior pediatric resident, anesthesia staff physician or resident, respiratory therapist, two critical-care trained nurses, pharmacist, pediatric intensive care physician or fellow, and a security officer. Our hospital has 166 inpatient beds with over 6000 admissions annually and a Medical Emergency Team (MET) that is a subset of the code blue team (respiratory therapist, one critical care trained nurse, and a pediatric intensive care physician or fellow) available 24/7. The MET members receive regular training and exposure to critically ill patients, and the MET is typically requested for deteriorating patients in an effort to avoid ICU admission and code blue events.

### Inclusion and exclusion criteria

To focus on non-acute HCP actions, all activations of the code blue system for patients outside acute care areas (Emergency Department, Intensive care units, Operating room) during the study period were reviewed. All code blue activations were included, even if it was subsequently determined that the incident was not life-threatening, defined as the absence of significant cardiac or respiratory compromise. This was done because the actions of non-acute HCPs were still considered relevant, presuming their impression of the situation was of a significantly deteriorating patient. Code blue events that were activated by, or occurred in the presence of the MET, were excluded from the final dataset, as MET presence confounds the outcome of interest (resuscitation performance of ward non-acute HCPs alone).

### Outcomes

The main outcomes of interest were the assessments and interventions (critical actions as outlined in life support algorithms) performed by non-acute HCPs prior to the arrival of the code blue team as documented in the patient chart (Table S1). These critical actions were adapted from those actions recommended in pediatric BLS and assessed in simulation studies [9,10,12,13]. An airway, breathing and circulation assessment was considered completed if the patency of the airway, the quality of respiratory effort, and a pulse check were documented, respectively. All other critical actions were considered completed if there was documentation regarding their performance. Examples of critical actions include assessment of each of airway, breathing, circulation (ABC) and level of consciousness; positioning the airway; suctioning the airway; applying oxygen; bag-valve-mask ventilation use (BVM); chest compressions; confirming/placing intravenous (IV) access; checking blood glucose level; and attaching monitors.

Code blue event characteristics and demographic information were collected to describe the patient and provider samples. Secondary outcomes (transfer to higher level of care, defibrillation or intubation prior to transfer, and survival to discharge) were also collected. Additionally, a secondary analysis associating critical action performance with the presence of established leadership was done. Leadership was considered ‘established’ if documentation listed one participant as directing the actions of other participants during the resuscitation prior to the arrival of the code blue team.

### Validity analysis

Given the anticipated limitations of retrospective chart data, two forms of concurrent criterion validity data were also collected for comparison. First, critical action performance data by the code blue team was collected for the final 18 months of the study analysis period. These data were seen as relatively high quality as the code blue team uses a standardized template with a dedicated pre-assigned team member to document during resuscitations. Second, a convenience sample of MET nurses, ward nurses, RRTs, and physicians all with a history of involvement with at least one code blue event were asked, ‘Imagine you were reviewing the last 10 code blue activations on the wards at [the hospital]. In the few minutes before the code team arrives, in how many of those 10 cases would you estimate the ward team has done the following: assess the airway for patency, assess breathing effort/effectiveness, assess circulation with a pulse check, assess all 3 of the above?’

### Data collection

All code blue activations have been prospectively tracked and recorded at the hospital since 2007 with clear identifying data since 2008. The study dataset consisted of all code blue activations occurring within the hospital but outside of intensive care units or emergency department settings. The hospital charts were reviewed and event demographic data as well as the outcome measures listed above were extracted using a standard case report template. Outcomes of interest were extracted from the earliest documentation that described actions leading up to and including the activation of the code blue until the arrival of the code blue team.

### Data analysis

All data were de-identified and transferred to a REDCap database [14,15]. The statistical plan was developed in consultation with a biostatistician. Descriptive statistics were tabulated for patient and event demographic details. Discrete variables were summarized using frequencies and percentages; continuous variables were summarized using means and standard deviations as well as medians with quartile 1 and quartile 3 (Q1, Q3). Performance of critical action was summarized using frequency and percentage. Perception of critical action performance was summarized using means and standard deviations.

Associations between the establishment of leadership and the performance of critical actions were measured using odds ratios (OR) estimated via logistic regression, together with 95% confidence intervals. In one instance, Firth logistic regression was performed due to difficulties encountered with convergence of the logistic regression algorithm, due to a zero cell count. All analyses were performed using R version 3.6.3[16].

## Results

There were 60 code blue activations identified during the study period. Two charts were unavailable due to ongoing legal proceedings and were therefore not included in the analysis. Fifty-eight code blue activations were included in the analysis

### Patient and event characteristics

Thirty-five percent (19/55) of code blue activations were for cardiopulmonary arrest, and more than half of activations (55%, 30/55) were for patients in respiratory distress or failure. Three code blue activations did not document the reason for the activation. The MET was activated prior to a code blue activation in 35% (20/57) of cases, with one instance where it was unclear from the documentation whether the MET was activated prior to the code blue activation. The MET itself activated the code blue in 17% (10/58) of cases for the purpose of intubation and mobilizing resources. Registered nurses (RNs) were the HCP most likely to be present prior to code blue activation (95%, 55/58) (Table 1).

**Table 1.** Demographics and Characteristics of Code Blue Activations.

	N = 58
Age, *years*	
median (Q1, Q3)	0.8 (0.3, 10.6)
Sex, *n (%)*	
Male	27 (46.6)
Female	30 (51.7)
Diagnostic categories, *n (%)*	
Respiratory	21 (36.2)
Infectious	6 (10.3)
Hematology/Oncology	6 (10.3)
Cardiac	4 (6.9)
Gastrointestinal	6 (10.3)
Neurology	5 (8.6)
Other	10 (17.2)
MET Prior Involvement, *n (%)*	
Active	8 (13.8)
Signed Off	21 (36.2)
Not involved prior to event	28 (48.3)
Date MET Signed Off, *days^a^*	*n = 21*
mean (SD) [range]	7.3 (12.7) [0.0, 51.0]
median (Q1, Q3)	3.0 (1.5, 6.5)
*C*ode Team Arrival Time, *mins^b^*	
mean (SD)	3.4 (2.4)
median (Q1, Q3)	3.0 (2.0, 5.0)
Code Activated Pre-arrest, *n (%)*	
Yes	38 (65.5)
No	18 (31.0)
MET Activated Prior to Code Blue, *n (%)*	
Yes	20 (34.5)
No	36 (62.1)
HCP Present Before Code Activation, *n (%)*	
RN	55 (94.8)
RT	5 (8.6)
Junior Resident	10 (17.2)
Senior Resident	9 (15.5)
Attending	9 (15.5)
MET	10 (17.2)
Unclear	1 (1.7)
Other^c^	4 (6.9)
Arrest Type – Reason for Code, *n (%)*	
Primary Cardiac Pulseless	1 (1.7)
Primary Unstable Bradycardia	4 (6.9)
Primary Respiratory Pulseless	14 (24.1)
Respiratory Distress with Pulse	16 (27.6)
Seizure	6 (10.3)
For Intubation on Floor	9 (15.5)
Other^d^	5 (8.6)

^a^Missing values from 6 cases

^b^Missing values from 2 cases

^c^OR staff, health care aide, care worker, sleep lab tech

^d^Apnea without pulse check, stroke, Anaphylaxis x 2, Drug reaction with possible Anaphylaxis

HCP, healthcare provider. MET, medical emergency team. RN, registered nurse. RT, respiratory therapist. SD, standard deviation. Q1, quartile 1. Q3, quartile 3.

Overall, 93% (54/58) of children survived to discharge from hospital with only 1 patient dying prior to transfer to PICU. There were no instances of defibrillation (Table 2). Of the 58 code blue activations, 26% (15/58) were deemed stable enough to remain on the pediatric ward following assessment and management by the code blue team (Table 2).

**Table 2.** Code Blue Outcomes.

	N = 58, (%)
Intubated On Floor	
Yes	13 (22.4)
No	44 (75.9)
Shocked On Floor	
Yes	0 (0)
No	57 (98.3)
Died On Ward	
Yes	1 (1.7)
No	56 (96.6)
Survived to Discharge	
Yes	54 (93.1)
No	4 (6.9)
Transferred to PICU	
Yes	41 (70.7)
No	16 (27.6)

### Critical action performance

As previously described, the 10 activations where the MET was present at the time of code blue activation were removed from the analysis of non-acute HCP performance. Forty-eight code blue activations were therefore analyzed for non-acute HCP critical action performance.

An airway assessment was documented in 33% (16/48) of cases, a respiratory assessment was documented in 69% (33/48) of cases, and a pulse assessment was documented in 29% (14/48) of cases. A full ‘ABC’ assessment was documented in 6% (3/48) of cases (Table 3). There were 63% (30/48) of patients that were clearly identified as either apneic or having unstable respirations, with the respiratory status of another 25% (12/48) being unclear, and an intervention of BVM ventilation was documented in 71% (34/48) of cases. Eleven children (23%, 11/48) were assessed as being pulseless or having bradycardia with poor perfusion. Chest compressions were performed on 29% (14/48) of children for whom a code blue was activated. Of those children, backboard use during compressions was documented in 29% (4/14) of cases and pulse checks for adequacy of compressions were performed in 14% (2/14) of cases (Table 3).

**Table 3.** Critical Action Performance.

	N = 48^a^, *(%)*
First Leader Training Level	
Attending	11 (22.9)
Senior Resident	5 (10.4)
Junior Resident	2 (4.2)
PICU Resident: Non-code Team	1 (2.1)
No Leader Identified	29 (60.4)
If Airway Assessed	
Stable	4 (8.3)
Unstable	11 (22.9)
Unclear	20 (41.7)
Not Performed	13 (27.1)
If Respiratory Effort Assessed	
Stable	2 (4.2)
Unstable	11 (22.9)
Apneic	19 (39.6)
Unclear	12 (25.0)
Not Performed	4 (8.3)
BVM Use for Apneic/Unstable Respiratory Effort	
Yes	34 (82.9)
No	7 (17.1)
If Circulation Assessed	
Pulse present	5 (10.6)
Poor perfusion with HR < 60	3 (6.4)
Pulse Absent	8 (17.0)
Unclear	12 (25.5)
Not Performed	19 (40.4)
If Pulse Absent or HR < 60 with Poor Perfusion	
Chest Compressions	14 (24.1)
Backboard Used for Compressions	4 (6.9)
Pulse Check with Compressions	2 (3.4)
Compression/Ventilation Ratio Appropriate	1 (1.7)
If LOC Assessed	
Awake	6 (12.5)
Unresponsive	20 (41.7)
Unclear	18 (37.5)
Not Performed	4 (8.3)
Blood Glucose Checked	
Yes	1 (2.1)
No	46 (97.9)

^a^Cases where the Medical Emergency Team activated the Code Blue were excluded to provide information on critical action performance by non-acute care healthcare providers

BMV, bag mask ventilation. HR, heart rate. LOC, level of consciousness

### Leadership

**Figure 1.**
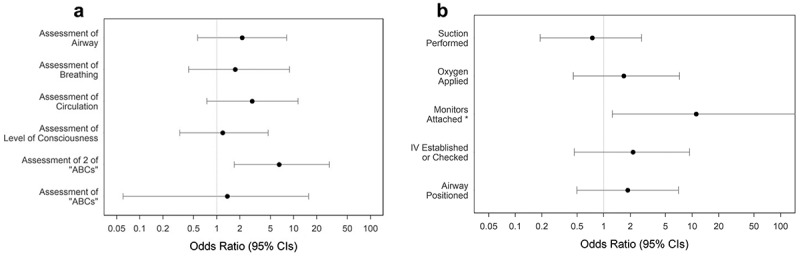
Analysis of the association of the presence of documented leadership with critical action performance. a, Leadership and performance of critical assessments. b, Leadership and performance of critical interventions.

Establishment of leadership was documented in 40% (19/48) of code blue activations prior to the code blue team arrival (Table 3). Established leadership prior to code blue team arrival was not statistically associated with individual assessments, but established leadership was associated with a partial assessment (OR 6.5, 95% CI 1.7–29.2, p = 0.01) (Figure 1a). The only intervention that was associated with documented leadership was attaching monitors (OR 11.1; 95% CI 1.3–1470, P = 0.03) (Figure 1b).

*Upper CI not shown as extends beyond 1000 CI, confidence interval. IV, intravenous. OR, odds ratio.

### Validity analyses

**Figure 2.**
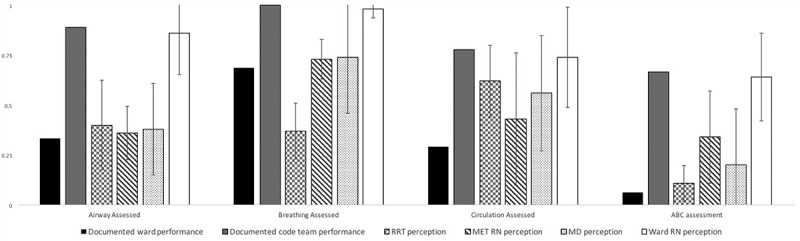
Comparison of documented critical action performance and perception of performance. Error bars indicated standard deviation.

Twenty providers (five each of ward nurses, MET nurses, physicians, and RRTs) were interviewed and provided their perceptions of critical action performance frequency prior to code blue team arrival. Also, critical action performance by the code blue team was extracted from nine events that occurred in the final 18-months of the study period. Both sets of data and the main study findings are shown in Figure 2.

MD, medical doctor. MET, medical emergency team. RN, registered nurse. RRT, registered respiratory therapist.

## Discussion

The goal of this study was to determine whether pediatric non-acute HCP performance during real-life significant patient deterioration was consistent with standard life support guidelines. While by no means definitive, our data lend further credibility to simulation-based findings that non-acute HCPs do not consistently complete critical life support assessment and intervention actions. Documentation of a full ‘ABC’ assessment was rare, present in 6% (3/48) of cases prior to the code blue team arrival. A pulse check was documented in less than 30% (14/48) of cases, whereas respiratory status was most likely to be assessed (33/48 [69%]). In addition to the challenges with performing full ‘ABC’ assessments, we also found deviations from life support guidelines for BLS interventions. Airway maneuvers, including positioning and suctioning, were each documented in less than half of cases despite respiratory concerns being the most common reason for code blue activation. Together, these results support the concern that provider performance is inconsistent with established life support guidelines in the real-life acute situations and suggest that current education models may be inadequate.

Our results lend credibility to previous simulation studies that show errors in BLS skills by both physicians and nurses during mock codes [9,10,17,18]. For example, Hunt et al. conducted 34 simulated pediatric resuscitation events in non-acute hospital areas and found that teams adhered to guidelines in their administration of BVM ventilation and chest compressions in 19% and 27% of cases, respectively [9]. The same study determined that only 25% of teams adhered fully to BLS guidelines. These simulation-based findings are comparable to our results and taken together suggest that non-acute HCPs may commonly deviate from life support guidelines in the real world. This is especially concerning given that first responders play a key role in improving outcomes for deteriorating patients[19]. BLS is often life-saving, but requires proper assessment to guide appropriate interventions[12]. Indeed, improving recognition of and response to deteriorating patients by non-acute care HCPs has been correlated with improved patient outcomes[20]. Importantly, the performance described in both the simulation and real-life experiences occur despite staff having received standard life support training courses[10]. For pediatric non-acute HCPs, however, resuscitation events are low frequency, high-stake situations. The low frequency of these events combined with BLS and PALS training requiring infrequent certification, often annually and every 2 years, respectively, likely leads to a degradation of skills. Studies have shown poor retention of knowledge and skills with the current resuscitation training approaches [21–24]. Declines in knowledge and technical skills have been shown to occur within months of PALS training [24,25]. Our results are therefore unsurprising, and the deviations from established resuscitation guidelines may reflect a systemic issue with regard to our current approaches to resuscitation training.

Our study supports the notion that novel educational approaches in resuscitation training are needed, as the potential for improved patient outcomes with better training is possible[26]. Recent research suggests that simulation education is effective in improving resuscitation skills in HCPs and is a crucial method for training non-acute HCPS, as clinical exposure to resuscitation situations is rare [27–31]. Specifically, deliberate practice techniques within simulation-based education, which address identified weaknesses with repetition and feedback immediately directed at those weaknesses, have garnered significant attention[32]. A curriculum designed on the principles of deliberate practice has been shown to be effective in improving the performance and retention of resuscitation skills [31,33]. Given the results of our study and simulation literature showing that non-acute HCPs often have difficulty applying BLS, there is a need for focused training to address these deficiencies. Our results also highlight that RNs are commonly, and often the only providers, present at the time of code blue activation. Accordingly, pediatric inpatient resuscitation education should include a focus on nurses.

Building on our data and existing literature, including current recommendations, an effective training program should include principles of deliberate practice, be context-specific or compatible, occur at regular intervals, and be interprofessional in nature with a focus on nurses[34]. Our institution has recently piloted a relatively low-resource program based on these principles with overall positive results in terms of perceived usefulness from participants[35]. We believe that there is a clear need for regular, brief, in-situ and context-specific simulation training focusing on skills that non-acute HCPs need to begin the resuscitation of deteriorating or arrested children.

Our study also suggests that in addition to developing new training modalities for resuscitation-specific knowledge and skills, there may be utility in focusing on improving leadership skills. Simulation studies have suggested that leadership training can lead to improved and sustained cardiopulmonary resuscitation performance [36–38]. Leadership training has been identified by the AHA as an essential component of resuscitation education[34]. Unfortunately, few residents feel confident enough in their skills to lead a resuscitation following traditional PALS training[24]. In our study, the association between the performance of the most critical actions and leadership has not reached statistical significance, but reviewing the odds ratio data in Figure 1 raises the possibility that small benefits with leadership may exist, and perhaps be detectable with a larger sample size. Nonetheless, developing leadership skills in non-acute HCPs may lead to improved assessment and initial management of the deteriorating pediatric patient. We suggest that future education curricula for non-acute care HCPs should include the development of leadership in addition to traditional technical skills and that more research is needed in this area.

One of the major challenges faced in our study was the variability and quality of documentation. While our institution has standardized documentation for the code blue team with a dedicated real-time documenter, there is no standard documentation prior to the arrival of the code blue team. Documentation was noted to be variable in content and in individuals performing the documenting. We were unable to reliably identify which non-acute HCP performed critical actions when they occurred, which may have been useful data. The limitations of resuscitation documentation are not unique to our study, however, as this is a recognized problem that occurs even with critical care trained HCPs using standardized documentation templates [39]. Ultimately, in our study, we acknowledge that issues with documentation affect the validity of our results as it is possible that we have captured a lack of documentation of critical actions rather than a lack of actual performance of critical actions.

Acknowledging this possibility, in our opinion, three sources of data lend credibility to the validity of the findings. First, the results are reasonably similar to published findings from controlled simulation environments with challenges in adhering to basic life support algorithms [9,10,17,18]. Second, a review of performance data in Figure 2 suggests that even the code blue team of critical care experts with prospective/designated documentation does not operate with perfect adherence. It would be expected that ad-hoc teams of ward providers would function well below the dedicated code blue team. Finally, although subjective, recalled estimates of critical action performance by local providers indicate that our study findings are not unreasonable. Critical action performance rates reported by respiratory therapists, physicians, and MET nurses tended to concur, and approximated the results obtained from our current study. Interestingly, reports by ward RNs were divergent and described ward provider rates that were higher and approximated those of the actual code blue team. Given that our study reveals that ward RNs are the predominant provider type present at the time of code blue activation, we surmise this finding may be due to known inaccuracies in self-assessment [40–42].

## Conclusions

Our study suggests that the performance of non-acute pediatric HCPs, including both nurses and physicians, often deviates from established resuscitation guidelines when managing a deteriorating patient. The findings support the notion that these providers require a different educational approach to develop and maintain the relevant resuscitation skills they would be expected to perform in their clinical environment. Our study also supports ensuring particular attention to training ward nurses, as they are by far the most common provider type present at the time of a code blue activation.

## Supplementary Material

Supplemental Material

## Comments

An abstract of this work was presented at the 2018 American Academy of Pediatrics National Conference & Exhibition, and subsequently published in the conference proceedings.
