# Supplementary material for: Pediatic code blue event anaylsis: Performance of non-acute health-care providers
Source: Med Educ Online. 2022 Aug 1;27(1):2106811. doi: 10.1080/10872981.2022.2106811 (PMC9347468; doi:10.1080/10872981.2022.2106811)
Supplement: Supplemental Material [file ZMEO_A_2106811_SM1722.docx]

| **Supplementary Table 1**. Critical Actions | |
| --- | --- |
| Assessments |  |
| Airway |  |
| Breathing |  |
| Circulation |  |
| Level of Consciousness |  |
| Interventions |  |
| Airway Positioned |  |
| Airway Suctioned |  |
| Oxygen Applied |  |
| IV Established/Checked |  |
| Monitors Attached |  |
